# Diagnostic and prognostic relevance of CP2c and YY1 expression in hepatocellular carcinoma

**DOI:** 10.18632/oncotarget.15462

**Published:** 2017-02-17

**Authors:** Ji Sook Kim, Seung Han Son, Min Young Kim, DongHo Choi, Ik-Soon Jang, Seung Sam Paik, Ji Hyung Chae, Vladimir N. Uversky, Chul Geun Kim

**Affiliations:** ^1^ Department of Life Science and Research Institute for Natural Sciences, College of Natural Sciences, Hanyang University, Seoul 04763, Korea; ^2^ Department of Pathology, Hanyang University College of Medicine, Seoul 04763, Korea; ^3^ Department of Surgery, Hanyang University College of Medicine, Seoul 04763, Korea; ^4^ Division of Bioconvergence, Korea Basic Science Institute, Daejeon 34133, Korea; ^5^ Department of Molecular Medicine, USF Health Byrd Alzheimer's Research Institute, Morsani College of Medicine, University of South Florida, Tampa, Florida 33612, USA

**Keywords:** hepatocellular carcinoma (HCC), CP2c, YY1, diagnosis, prognosis

## Abstract

Recent studies have demonstrated an oncogenic role of the transcription factor (TF) CP2c in hepatocellular carcinoma (HCC) based on a strong correlation between CP2c expression, tumor grade, and aggressiveness. We recently found that CP2c directly interacts with another TF, YY1, which is also overexpressed in multiple cancers, including HCC. To evaluate if these proteins are co-regulated in carcinogenesis, we analyzed the expression of CP2c and YY1 in HCC (*n* = 136) tissues and examined the correlation between their expression and clinicopathological characteristics of HCC. Receiver operating characteristic analysis exhibited the validity of CP2c and nuclear YY1 expression as a diagnostic factor in HCC tissues. High expression of CP2c was significantly correlated with patient age, and higher histological grade, American Joint Committee on Cancer (AJCC) stage, and small and large vessel invasion in HCC tissues, whereas high expression of nuclear YY1 was significantly associated with higher AJCC stage and small vessel invasion. In univariate and multivariate analyses, high expression of CP2c was significantly correlated with disease free survival (DFS), indicating that CP2c expression is an independent prognostic factor for DFS in HCC patients. Patients with high expression of both CP2c and nuclear YY1 usually had a shorter median survival time and worse DFS prognosis than other patients, suggesting that combined detection of CP2c and nuclear YY1 is a useful prognostic marker in HCC patients.

## INTRODUCTION

Transcription factor (TF) CP2c (also known as TFCP2, α-CP2, LSF, and LBP-1c) was first identified as a transcriptional activator of the α-globin gene in erythroid cells [1–2]. There are six CP2 isoforms in humans (LBP-1a, -1b, -1c, -1d, -9 and LBP-32) and four in mice (CP2a, CP2b, CP2c and CRTR-1) [3–4]. CP2c, a member of the CP2 family of proteins, participates in diverse processes including hematopoiesis, immune response, cell cycle, and neural development by regulating the expression of specific target genes [5]. Interactions between CP2c and other isoforms of the CP2 family as well as various partner proteins, allow for the regulation of specific target genes in different cellular environments [6–11]. Recent studies have demonstrated that CP2c has an oncogenic role in hepatocellular carcinoma (HCC) [12–15]. First, it was found to confer 5-FU resistance to HCC cell lines by activating the expression of *thymidylate synthase* (*TS*) gene [16–18]. Second, CP2c was shown to activate *osteopontin* (*OPN*) and *matrix metalloproteinase-9* (*MMP-9*) expression and regulate invasion, metastasis, and angiogenesis of HCC cells [19–20]. It was also shown that CP2c could be activated by Notch signaling and promoted HepG2 cell proliferation and invasion [12]. Indeed, CP2c expression has been shown to be significantly upregulated in HCC, cervical cancer, and colorectal carcinoma [12, 21–22].

We recently found that CP2c and YY1 interact directly with each other, and their expression is reciprocally regulated in the spermatogonial stem cells and different stages of cells during spermatogenesis [23–24]. Like CP2c, YY1 is a ubiquitously expressed TF involved in diverse biological processes, such as embryogenesis, differentiation, proliferation, and cancer progression [25–28]. YY1 is well known for its dual roles in regulating gene expression, either as an activator or repressor, depending on the chromatin remodeling complexes it is recruited to [29]. There is increasing evidence that YY1 is important in cancer development. Overexpression of YY1 has been observed in various cancers, including prostate cancer, ovarian cancer, and colon cancer [30–32]. The role of YY1 in cancer is due to its ability to modulate many genes involved in cancer development and progression, such as *c-myc*, *c-fos*, *ERBB2*, *CEBPA*, and *p53* [26, 33–34]. Recent studies showed that YY1-mediated epigenetic silencing of tumor-suppressive microRNAs activated hepatocarcinogenesis and melanoma tumorigenesis [35–36]. Although the expression and regulatory roles of CP2c and YY1 have been reported individually for several types of cancer, co-regulation of these proteins in carcinogenesis has not been specifically explored as of yet.

In this study, we analyzed the expression of CP2c and YY1 in normal liver, adjacent noncancerous liver, and HCC tissues and examined the correlation between their expression and clinicopathological characteristics of HCC. In addition, the significance of combined detection of CP2c and YY1 expression as a prognostic factor of HCC outcome was evaluated using various statistical methods such as receiver operating curve analysis (ROC), survival analysis, and univariate and multivariate analyses.

## RESULTS

### Differential expression of CP2 family, CP2c, and YY1 proteins in liver tissues

**Figure 1:**
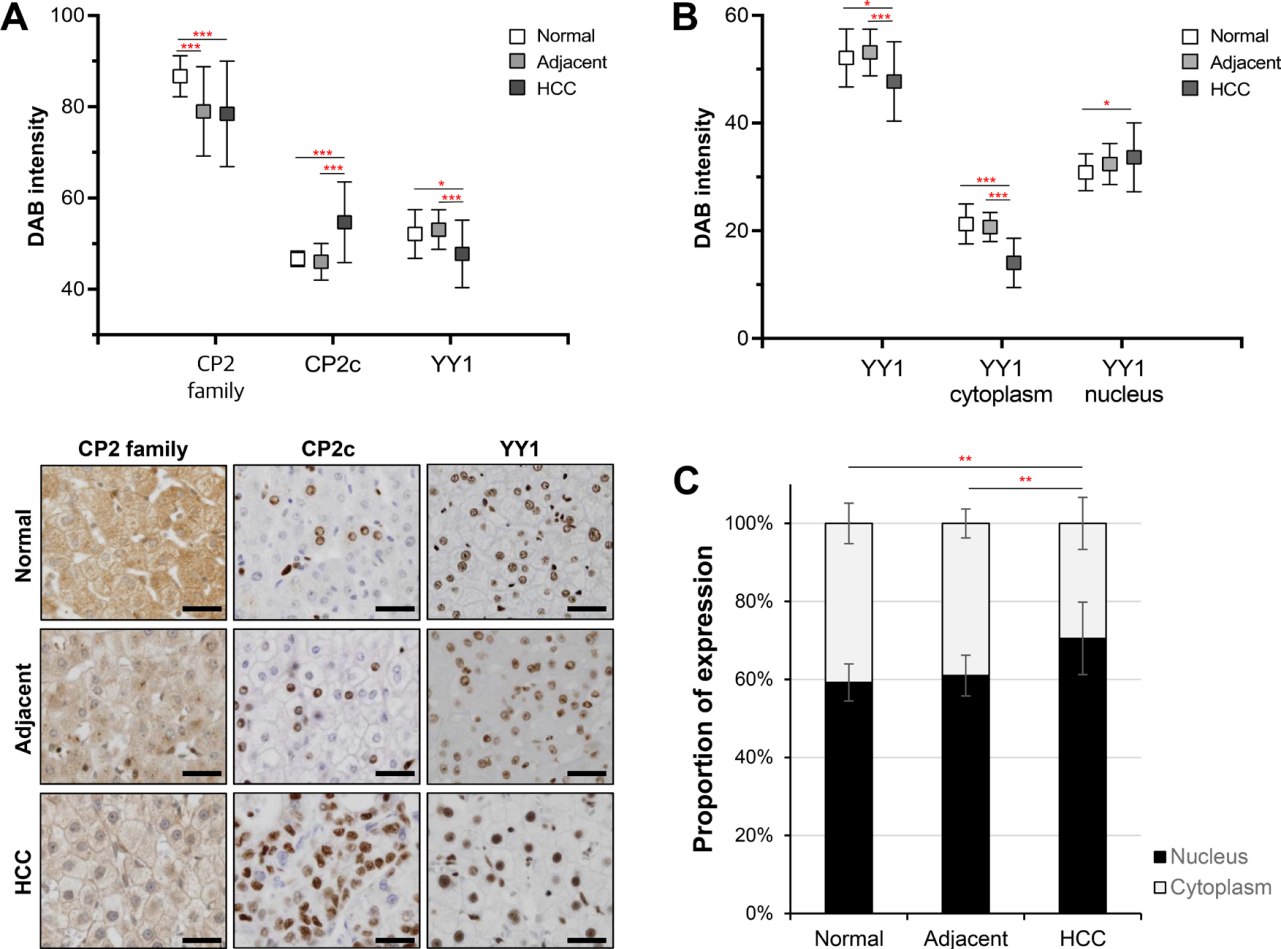
Expression of CP2 family, CP2c, and YY1 proteins in liver tissues (**A**) Protein expression of CP2 family, CP2c, and YY1 proteins was quantitatively analyzed by the TissueFAXS system in normal liver (*n* = 16), adjacent noncancerous (*n* = 48), and HCC (*n* = 136) tissues (upper panel). Representative images of immunohistochemical staining (bottom), Scale bar = 50 μm. (**B**) Expression level of YY1 in different cellular compartments was examined using a TissueFAXS scanning and analysis system. (**C**) The proportion of nuclear YY1 expression was significantly higher in HCC samples than normal and adjacent noncancerous tissues. *P < 0.05, **P < 0.01, ***P < 0.001 (Student's *t*-test).

The expression and cellular distribution of CP2 family (CP2a, CP2b, and CP2c), CP2c, and YY1 proteins in normal human liver (*n* = 16), adjacent noncancerous (*n* = 48) and HCC (*n* = 136) tissue samples were analyzed by immunohistochemistry (IHC) and quantified by TissueFAXS system (TissueGnostics, Vienna, Austria) (see Materials and Methods). CP2c was expressed at significantly higher levels in HCC tissues than normal liver or adjacent noncancerous (ADJ) liver tissues, whereas YY1 was expressed at lower levels in HCC compared with normal or noncancerous liver tissues (Figure 1A). The expression of CP2 family proteins was higher in normal liver than ADJ or HCC tissue samples. Similar expression patterns of CP2 family, CP2c, and YY1 proteins were also observed both in western blot and in reverse transcriptase-quantitative PCR (RT-qPCR) analyses of the two selected HCC samples in the tissue array, along with the matched ADJ noncancerous liver tissues derived from the same patients and two non-matched normal liver samples (Supplementary Figure 1). Therefore, these observations suggest that our quantitative IHC data are reliable.

CP2 family proteins were mainly localized in the cytoplasm and weakly in the nucleus, whereas CP2c and YY1 proteins were present in the nucleus (Figure 1A, Bottom). However, YY1 expression was also detected, albeit at very weak levels, in the cytoplasm of normal or ADJ liver tissue samples. To compare the cellular distribution of YY1 expression, we also measured the immunoreactivity of YY1 in the nucleus and cytoplasm by TissueFAXS system. Nuclear YY1 expression was significantly higher in HCC samples than in normal or ADJ liver tissues (Figure 1B). Furthermore, the extent of nuclear YY1 expression was about 10% higher in HCC than in normal or ADJ samples (Figure 1C). Expression levels and frequencies of expression of CP2 family, CP2c and YY1 proteins in individual samples from the normal/ADJ and the HCC groups are shown in Supplementary Figure 2. These data indicate that CP2 family and YY1 proteins, which are components of a joint TF network, are differentially expressed in noncancerous liver and HCC tissues, suggesting that they may play a coregulatory role in HCC development and/or progression.

### Evaluation of CP2 family, CP2c, and YY1 proteins as diagnostic biomarkers of HCC

**Figure 2:**
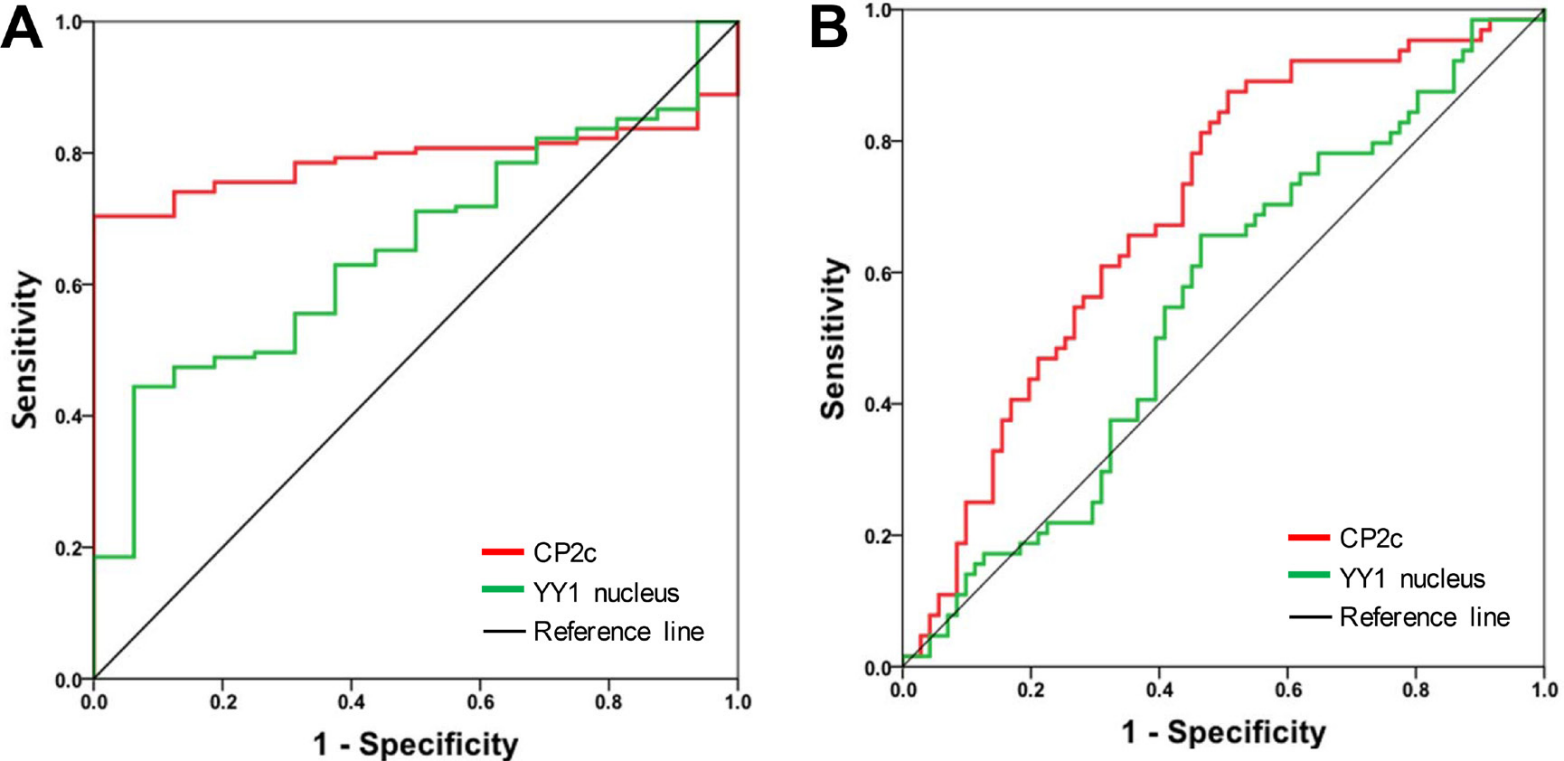
Receiver operating characteristic (ROC) curves of CP2c and nuclear YY1 expression as diagnostic markers for HCC (**A**) ROC curves for CP2c and nuclear YY1 expression in normal liver versus HCC tissue samples. (**B**) ROC curves to determine the optimal cut-off values for CP2c and nuclear YY1 expression according to disease free-survival in 116 HCC patients.

To evaluate the diagnostic significance of CP2c and YY1, we constructed receiver operating characteristic (ROC) curves by plotting sensitivity versus specificity (Figure 2). The areas under the ROC curves (AUCs) for CP2c and nuclear YY1 expression were 0.791 (*P* < 0.01) and 0.657 (*P* = 0.040) for discriminating HCC patients and normal groups, respectively (Figure 2A). The AUC of CP2 family, YY1, and cytoplasmic YY1 protein expression was not significant (Supplementary Table 1). These data indicate that CP2c expression and nuclear YY1 expression may be diagnostic markers of HCC. According to Sox et al. [37], an AUC equal to or greater than 0.7 indicates the acceptance of using a marker in diagnosis. Therefore, we focused on CP2c expression and nuclear YY1 expression and calculated the optimal cut-off value of CP2c and nuclear YY1 expression to evaluate the relationship between disease-free survival (DFS) rate and marker gene expression in 116 HCC patients (Figure 2B & panel B of Supplementary Table 1). ROC exhibited that CP2c and nuclear YY1 expression both may have significant correlation with regard to DFS (AUC = 0.696 for CP2c, AUC = 0.553 for YY1).

### Relationship between CP2c and nuclear YY1 expression and clinicopathological characteristics of HCC

To evaluate the correlation of CP2c expression and nuclear YY1 expression with tumor biology, 136 HCC samples were classified as having low or high CP2c and nuclear YY1 expression based on the optimal cut-off points calculated from ROC analysis using Youden index, as described previously [38] (Supplementary Table 1). The representative images of low or high expression of CP2c and nuclear YY1 were shown in Supplementary Figure 3. According to this criteria, we analyzed the relationship between CP2c and nuclear YY1 expression and clinicopathological characteristics (Table 1). Elevated expression of CP2c in HCC tissues was significantly correlated with age (*P* = 0.013), higher histological grade (*P* = 0.024), AJCC stage (*P* = 0.005), and small and large vessel invasion (*P* = 0.009 and *P* = 0.038, respectively). It is noted that CP2c expression was also significantly correlated with the individually grouped histological grade and AJCC stage (Supplementary Table 2). High expression of nuclear YY1 was also associated with higher AJCC stage (*P* = 0.002) and small vessel invasion (*P* = 0.01) (Tables 1 and Supplementary Table 2). However, we did not observe any significant relationships between the CP2c expression and other clinicopathological variables or nuclear YY1 expression and other clinicopathological variables (Table 1).

**Table 1:** Correlation of CP2c expression, nuclear YY1 expression, and clinicopathological characteristics in HCC

Characteristics		CP2c		*P* value(*x*^2^ test)	YY1 nucleus		*P* value(*x*^2^ test)
		Low	High		Low	High	
Age				0.013			0.515
	< 57	14 (20.0%)	56 (80.0%)		29 (41.4%)	41 (58.6%)	
	≥ 57	26 (39.4%)	40 (60.6%)		31 (47.0%)	35 (53.0%)	
Gender				0.059			0.822
	Female	14 (42.4%)	19 (57.6%)		14 (42.4%)	19 (57.6%)	
	Male	26 (25.2%)	77 (74.8%)		46 (44.7%)	57 (55.3%)	
HBsAg status				**0.134**			0.553
	Negative	13 (39.4%)	20 (60.6%)		16 (48.5%)	17 (51.5%)	
	Positive	26 (25.7%)	75 (74.3%)		43 (42.6%)	58 (57.4%)	
Histological grade				**0.024**			0.234
	G1 & G2	23 (39.7%)	35 (60.3%)		29 (50.0%)	29 (50.0%)	
	G3 & G4	17 (21.8%)	61 (78.2%)		31 (39.7%)	47 (60.3%)	
AJCC stage				**0.005**			**0.002**
	I	28 (40.0%)	42 (60.0%)		40 (57.1%)	30 (42.9%)	
	II ∼ IV	12 (18.2%)	54 (81.8%)		20 (30.3%)	46 (69.7%)	
Tumor size				0.600			0.373
	< 5 cm	27 (27.8%)	70 (72.2%)		45 (46.4%)	52 (53.6%)	
	≥ 5 cm	12 (32.4%)	25 (67.6%)		14 (37.8%)	23 (62.2%)	
Small vessel invasion				**0.009**			**0.010**
	Absent	30 (38.5%)	48 (61.5%)		42 (53.8%)	36 (46.2%)	
	Present	10 (17.5%)	47 (82.5%)		18 (31.6%)	39 (68.4%)	
Large vessel invasion				0.038*			0.258
	Absent	39 (32.8%)	80 (67.2%)		55 (46.2%)	64 (53.8%)	
	Present	1 (6.2%)	15 (93.8%)		5 (31.2%)	11 (68.8%)	
Perineural invasion				1.000*			0.629*
	Absent	39 (29.8%)	92 (70.2%)		59 (45.0%)	72 (55.0%)	
	Present	1 (25.0%)	3 (75.0%)		1 (25.0%)	3 (75.0%)	
Focality				0.158			0.306
	Single	36 (32.1%)	76 (67.9%)		52 (46.4%)	60 (53.6%)	
	Multiple	4 (17.4%)	19 (82.6%)		8 (34.8%)	15 (65.2%)	

*P* value < 0.05 marked in bold font shows statistical significance.

*Fisher's exact test

### Prognostic significance of CP2c and nuclear YY1 expression in HCC patients

**Figure 3:**
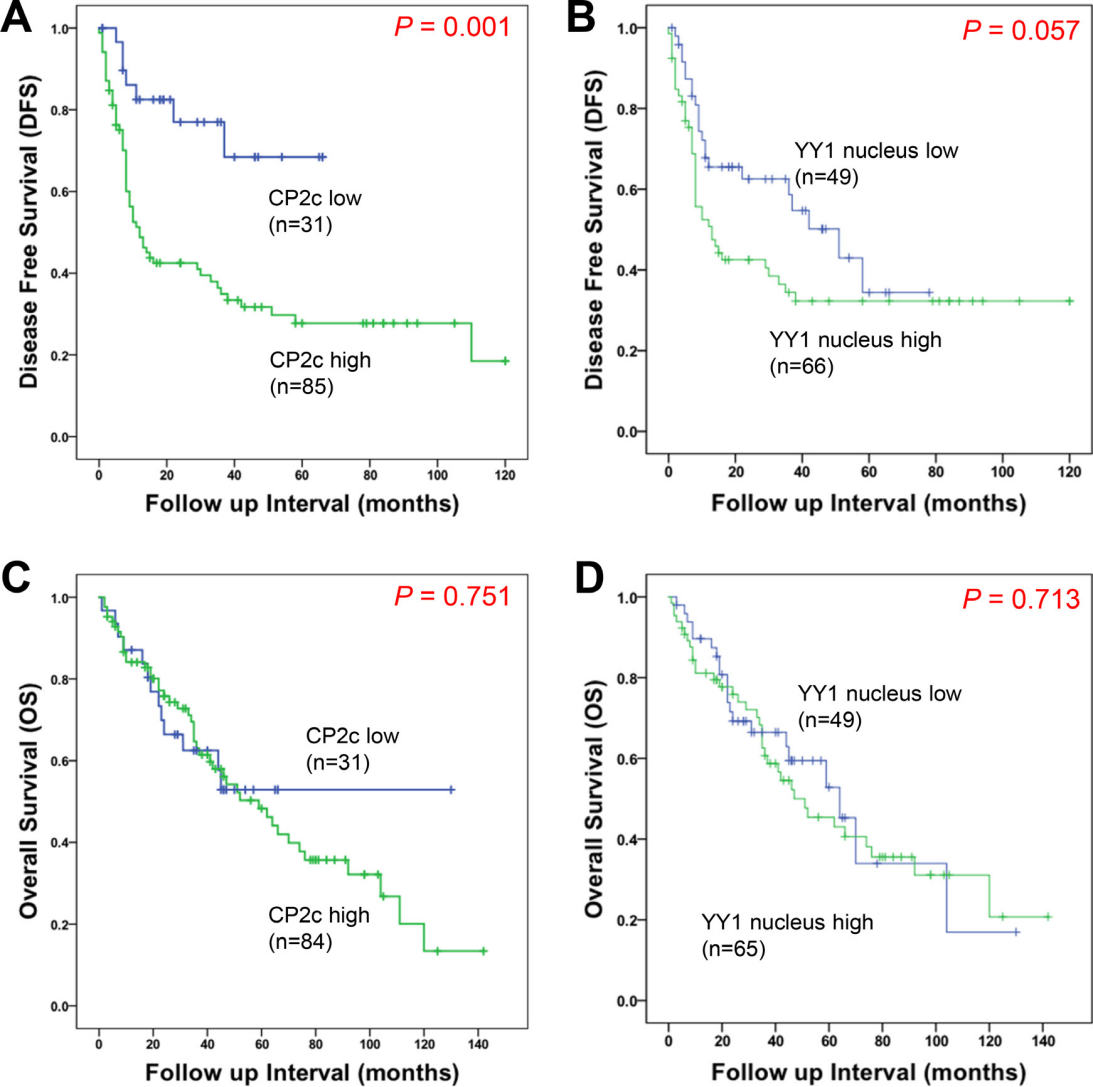
Kaplan-Meier survival curves showing the correlation between marker expression and disease-free survival (DFS) or overall survival (OS) (**A** and **B**) Kaplan-Meier curves for disease-free survival of HCC patient according to CP2c (A) or nuclear YY1 (B) expression. (**C** and **D**) Kaplan-Meier curves for overall survival of HCC patient according to CP2c (C) or nuclear YY1 (D) expression.

To estimate the relationship between CP2c expression and patient survival and nuclear YY1 expression and patient survival, we performed Kaplan-Meier curve analyses for DFS and overall survival (OS). Patients with high expression levels of CP2c or nuclear YY1 usually had a shorter median survival time and worse DFS prognosis than those with low CP2c or low nuclear YY1 expression levels (Figure 3A and 3B). The median DFS of patients with high CP2c expression was 12.1 months (95% CI 7.68 – 16.51), while the median DFS of patients with the high expression of nuclear YY1 was 12.76 months (95% CI 5.57 – 19.96), compared to the 29.33 months (95% CI 8.37 – 50.29) for all patients. However, median OS was not significantly different between the two expression groups for either marker (Figure 3C and 3D). To further investigate prognostic factors for poor HCC outcome, univariate and multivariate analyses were performed. In univariate analysis, DFS was correlated significantly with histological grade, AJCC stage, tumor size, vessel invasion, perineural invasion, as well as high expression of CP2c (Table 2). High expression of nuclear YY1 was associated with DFS, but the association was not statistically significant (*P* = 0.058). Multivariate analysis revealed that large vessel invasion and high expression of CP2c were independent prognostic factors for DFS in HCC patients.

**Table 2:** Univariate and multivariate cox regression analysis of prognostic factors for DFS in HCC

Variables	Univariate analysis		*P* value	Multivariate analysis		*P* value
	HR	95% CI		HR	95% CI	
Age	0.79	0.48–1.29	0.340	1.37	0.75–2.51	0.312
Gender	0.51	0.26–1.01	0.052	0.72	0.34–1.51	0.380
HBsAg status	1.07	0.60–1.92	0.811	1.06	0.52–2.17	0.871
Histological grade	1.72	1.02–2.87	**0.037**	1.27	0.71–2.30	0.419
AJCC stage	3.72	2.21–6.30	**< 0.01**	1.78	0.63–4.99	0.276
Tumor size	1.89	1.10–3.24	**0.021**	1.80	0.99–3.27	0.056
Small vessel invasion	3.93	2.36–6.57	**< 0.01**	1.66	0.61–4.53	0.322
Large vessel invasion	6.72	3.34–13.53	**< 0.01**	3.05	1.35–6.89	**0.007**
Perineural invasion	3.30	1.01–10.80	**0.048**	1.25	0.31–5.03	0.753
Focality	1.69	0.93–3.07	**0.085**			
CP2c	3.53	1.60–7.76	0.002	3.24	1.26–8.33	0.015
YY1 nucleus	1.62	0.97–2.70	0.058	0.94	0.51–1.72	0.832

*P* value < 0.05 marked in bold font shows statistical significance.

Abbreviations: HR, hazard ratio; CI, confidence interval.

**Figure 4:**
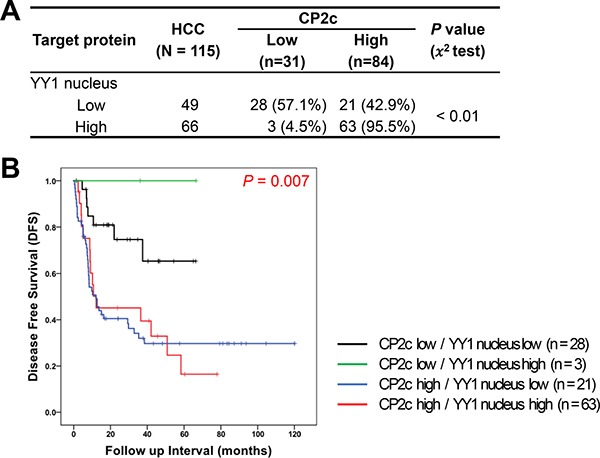
Prognostic significance of combined detection of high CP2c and nuclear YY1 expression in HCC patients (**A**) Association between CP2c and nuclear YY1 expression in HCC. (**B**) Kaplan-Meier curves for disease-free survival of HCC patients according to the combination of CP2c and nuclear YY1 expression.

To investigate the coregulatory role of CP2c and YY1 in HCC progression, we compared the combined expression pattern of CP2c and nuclear YY1 between normal or ADJ liver and HCC tissue samples. Among normal or ADJ samples (*n* = 59), the proportions of samples in each of the four possible expression groups were as follows: CP2c low/nuclear YY1 low (47.5%), CP2c low/nuclear YY1 high (44%), CP2c high/nuclear YY1 low (5.1%), and CP2c high/nuclear YY1 high (3.4%). For the HCC samples (*n* = 115), the proportions were as follows: CP2c low/nuclear YY1 low (24.3%), CP2c low/nuclear YY1 high (2.6%), CP2c high/nuclear YY1 low (18.3%), and CP2c high/nuclear YY1 high (54.8%) expression. These data suggest that high CP2c expression is a key diagnostic marker of HCC, and that combined detection of CP2c and nuclear YY1 may allow more useful diagnosis of HCC patients than either marker alone. We next analyzed the DFS rate in 115 HCC patients according to combined CP2c and nuclear YY1 expression. When the expression of CP2c was high, expression of nuclear YY1 was also significantly increased in the HCC patients (Figure 4A), whereas survival time decreased (Figure 4B). Interestingly, CP2c high/nuclear YY1 low and CP2c high/nuclear YY1 high expression groups had a similar median DFS (12.23 and 12.10 months, respectively); however, the 95% CI were significantly different (0.00 – 48.71 vs 6.37 – 17.83; *P* = 0.007). These data suggest that high expression of CP2c and nuclear YY1 is important for the HCC progression and poor outcome of the HCC patients.

## DISCUSSION

In this study, we examined the correlation between the expression of CP2c and YY1 and the clinicopathological characteristics of HCC to verify the relevance of CP2c and YY1 in HCC progression. We found that oncogenic CP2c expression by itself is a key diagnostic factor for HCC and that combined expression of CP2c and nuclear YY expression might be a useful prognostic factor for HCC.

CP2c expression was elevated in HCC tissues compared to normal and adjacent tissues, in concordance with previous findings [13, 17]. CP2c expression showed good discriminatory power with regard to DFS by ROC analysis and survival curve (Figures 2 and 3), and high expression of CP2c in HCC tissues was significantly correlated with higher histological grade, AJCC stage, and small and large vessel invasion (Table 1). In univariate and multivariate analyses, high expression of CP2c was significantly correlated with DFS, indicating that CP2c expression is an independent prognostic factor for DFS in HCC (Table 2). Our finding that CP2c expression is a key diagnostic marker of HCC is consistent with previous reports [12–13]. In addition, when we analyzed TCGA gene set (442 patient samples) in the public database (http://www.cbioportal.org), total CP2c gene alteration, including mRNA expression, copy number variation (CNV), and point mutation, was significantly correlated with DFS (*P* = 0.017). However, when individual CP2c gene alterations were analyzed by Kaplan-Meier analysis and Cox-regression, only CP2c mRNA upregulation showed significant correlation with DFS (*P* = 0.025 and *P* = 0.028, respectively) (data not shown).

Although *YY1* was proposed to promote hepatocellular carcinogenesis and inhibit cellular differentiation in HCC cell lines [34], and a significant increase in the nuclear YY1 protein expression was reported in HCC patients [39], nuclear YY1 expression has not previously been examined as a potential diagnostic factor for HCC. We found that the expression of nuclear YY1 was significantly higher in HCC samples than in normal or ADJ liver tissues (Figure 1). Indeed, ROC curve of the nuclear YY1 expression shows possibility as a diagnostic factor of HCC (AUC = 0.657) (Figure 2A and Supplementary Table 1), even though an AUC equal to or greater than 0.7 is used as an indicator of the acceptance of using a marker in diagnosis [37]. High nuclear YY1 expression showed significant association with higher AJCC stage and small vessel invasion (Table 1), a shorter median survival time and worse DFS prognosis (Figure 3). However, nuclear YY1 by itself was not strong enough to be used as a prognostic factor for DFS in HCC patients in either univariate or multivariate analyses (Table 2). In contrast, the combined high expression levels of both CP2c and nuclear YY1 might be a prognostic factor for the HCC patients, In fact, survival time was significantly lower in the HCC patients with high expression levels of both CP2c and nuclear YY1 (Figure 4) relative to the other expression groups. In addition, upregulation of the CP2 expression was associated with high expression of nuclear YY1 (Figure 4A). Therefore, our data indicate that nuclear *YY1* expression level is positively correlated with the CP2c expression and HCC progression, although nuclear YY1 by itself is not a prognostic factor in HCC. However, it is noted that the minor difference in the levels of nuclear YY1 expression between ADJ and HCC should be confirmed in subsequent studies by other methods, although our quantification of IHC images by Tissue FAXS system is quite reliable.

We noted that HCC patients with low CP2c expression and high levels of nuclear YY1 expression levels showed a good prognosis, whereas those with high CP2c expression had a bad prognosis regardless of the nuclear YY1 expression level (Figure 4), suggesting that CP2c is a driver of the HCC progression. Importantly, in patients with high CP2c expression, high nuclear YY1 expression was more significant than low nuclear YY1 expression in 95% confidence interval (Figure 4). Thus, our data suggest that the combination of high CP2c and nuclear YY1 expression is a useful prognostic marker of HCC. Meanwhile, it is noteworthy that the elevated expression of both of these factors in malignant HCCs is not consistent with our recent finding that CP2c and YY1 interact directly with each other and show functional cross-antagonism by mutual suppression of each other's activities [23, Kim et al., unpublished data]. Here, YY1 suppresses CP2c transcriptional activity by direct interaction with the DNA-bound CP2c, whereas CP2c indirectly suppresses YY1 transcriptional activity by promoting the degradation of the nuclear YY1 protein in a non-DNA bound state via the 20S proteasome pathway. This may be because the CP2c-YY1 crosstalk machinery is deregulated during the HCC progression. Obviously, further research is required to evaluate this hypothesis.

Because YY1 is overexpressed in many cancers, YY1 is considered to be a potential novel prognostic marker and therapeutic target [40–42]. However, as data on its prognostic significance has become available for more human cancers, YY1′s role in tumor progression has become more controversial [32]. Why YY1′s correlation with clinical outcomes is inconsistent among different cancers is unknown. One plausible reason is that YY1 regulates both cell proliferation and apoptosis, and in any given tumor, it may regulate one process more than the other by preferential interaction with available proteins depending on cellular contexts. For example, YY1 suppresses cell invasion and metastasis by downregulating MMP10 expression [43] and increases apoptosis through *BAX* activation in pancreatic cancer cells, suggesting that YY1 functions as a tumor suppressor [44]. YY1 is also known to antagonize *p53* through distinct mechanisms [45–46], and its inhibition may restore *p53* anti-tumor activity. However, the mechanisms underlying YY1 expression in these various cancers are unclear.

**Figure 5:**
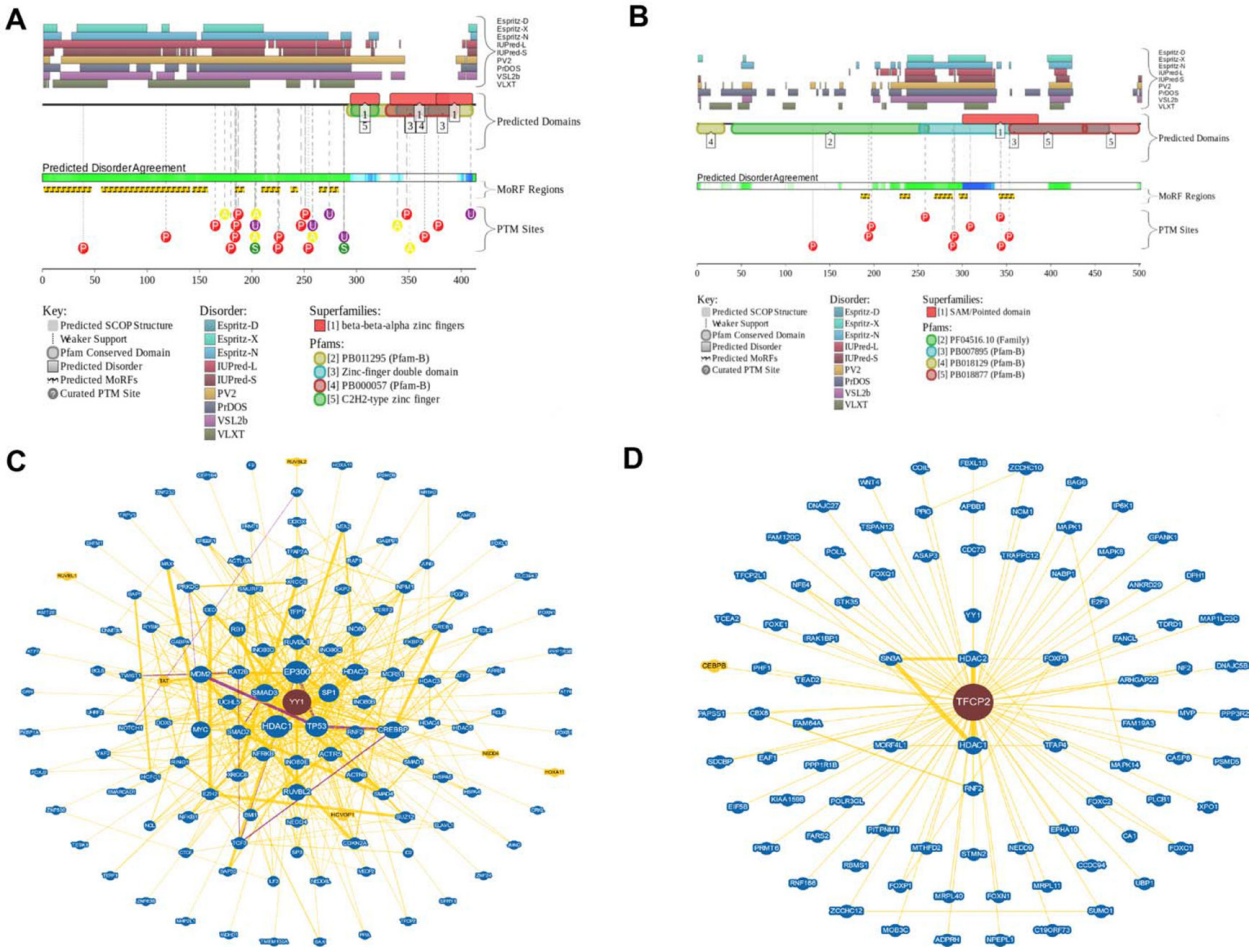
Functional disorder in YY1 and CP2c (**A** and **B**) Evaluation of the functional intrinsic disorder propensity of human YY1 (UniProt ID: P25490, plot A) and CP2c (UniProt ID: Q12800, plot B) analyzed by the D2P2 database (http://d2p2.pro/) [53]. Top nine colored bars represent location of disordered regions predicted by different disorder predictors (Espritz-D, Espritz-N, Espritz-X, IUPred-L, IUPred-S, PV2, PrDOS, PONDR^®^ VSL2b, and PONDR^®^ VLXT, see keys for the corresponding color codes). Green-and-white bar in the middle of the plot shows the predicted disorder agreement between these nine predictors, with green parts corresponding to disordered regions by consensus. Yellow bar shows the location of the predicted disorder-based binding site (MoRF region), whereas differently colored circles at the bottom of the plots show locations of various posttranslational modifications. (**C** and **D**) Analysis of the interactivity of human YY1 (plot C) and CP2c (plot D) using the BioGRID database containing information on published protein interactions [54]. Yellow nodes denote interactors in different organism, whereas blue nodes indicate interactors in same organism.

Another important question is related to the molecular basis of the exceptional polyfunctionality of YY1 and CP2c proteins. In fact, it was already emphasized that CP2c is involved in regulation of several important processes, such as cell cycle, hematopoiesis, immune response, and neural development by controlling expression of corresponding genes [5]. Furthermore, the ability of CP2c to be engaged in physical interaction with various partner proteins further increases functional complexity of this protein [6–11]. Similarly, YY1 has multiple critical roles in various biological processes, ranging from cell proliferation to cell differentiation, and from cancer progression to embryogenesis [25–28]. It is likely that the highly disordered nature of these two TFs hold an answer to the question on their polyfunctionality. In fact, earlier studies revealed that eukaryotic TFs typically do not have unique 3D structures, being characterized by high levels of intrinsic disorder [47–49]. Furthermore, polyfunctionality and high binding promiscuity are considered as characteristic features of many intrinsically disordered proteins [50–52]. In agreement with these earlier observations, Figure 5 shows that both YY1 and CP2c are predicted to be highly disordered and are characterized by very well developed interaction networks. In fact, according to the D2P2 analysis (http://d2p2.pro/) [53] (Figure 5A and 5B), very significant parts of YY1 and CP2c are expected to be disordered. Furthermore, disordered regions of both proteins are heavily decorated with multiple sites of various posttranslational modifications and are expected to have several disorder-based binding sites, molecular recognition features (or MoRFs), supporting the ability of these proteins to be involved in highly regulated and promiscuous interactions. Both high interactability of YY1 and CP2c and their ability to interact with each other is supported by the results of the BioGRID analysis (which is a public database, the biological general repository for interaction datasets that represents information on the published protein interactions) [54] (see Figure 5C and 5D). According to this analysis, human YY1 and CP2c are located at the center of well-elaborated interaction networks (see Figure 5C and 5D) that include 505 interactions between 129 different interactors and 109 interactions between 88 different interactors for YY1 and CP2c, respectively.

In conclusion, our data suggest that CP2c expression correlates with HCC initiation and progression, and that combined detection of high nuclear YY1 and high CP2c expression shows a poor outcome in the HCC patients. Thus, oncogenic CP2c expression by itself might be a key diagnostic factor for HCC, while combined CP2c and nuclear YY1 expression may be a useful prognostic factor for HCC.

## MATERIALS AND METHODS

### Patients and specimens

Paraffin-embedded tumor tissues were obtained from 136 primary hepatocellular carcinoma patients who underwent surgical resection between 2002 and 2013 at Hanyang University Hospital, Seoul, Korea. All study participants or their legal guardians provided informed written consent prior to study enrollment. Hematoxylin-eosin (H&E) slides, pathology reports, and other medical records were collected and reviewed to confirm the diagnoses as well as to obtain clinicopathological data about the tumors, such as age, gender, HBs Ag status, histological grade, AJCC stage, primary tumor size, small or large vessel invasion, perineural invasion, multi-focality, and clinical outcome. Clinicopathological characteristics are summarized in Supplementary Table 3. Median follow-up period was 27 months (range: 1–120 months) for DFS and 40 months (range: 1–120 months) for OS. Forty-eight adjacent liver tissue samples were obtained from the above-mentioned group. Sixteen normal liver tissue samples were also obtained from patients who underwent surgical resection for blunt trauma or benign neoplasm. This study was approved by the Institutional Review Board of Hanyang University Hospital (IRB file No. 2015-12-020-001).

### Construction of a tissue microarray

H&E stained slides made from the paraffin-embedded blocks were used to define the most morphologically representative, well fixed, and non-necrotic areas. Single tissue cores (2.0 mm in diameter) were punched from each paraffin block and assembled into a recipient paraffin block using a tissue microarray (TMA) instrument (AccuMax Array, ISU ABXIS, Seoul, Korea). TMA blocks contained 16 normal liver tissue samples, 48 non-neoplastic adjacent liver tissue samples, and 136 HCC tissue samples.

### Immunohistochemistry and evaluation of immunoreactivity value

Expression of CP2 family proteins as a whole, CP2c in particular, and YY1 was analyzed by IHC staining of TMA slides. Tissue sections (4-μm thick) were deparaffinized with xylene and then rehydrated with an ethyl alcohol series. Antigen retrieval was performed by autoclaving the samples for 30 min in 10 mM sodium citrate buffer (pH 6.0). Endogenous peroxidase activity was blocked by incubating sections with 3% hydrogen peroxidase solution for 15 min. Sections were incubated with rabbit polyclonal anti-CP2 family antibody (homemade Ab that could react with all CP2 isoforms, Cosmogentec), mouse polyclonal anti-CP2c antibody (610818, BD Biosciences), and rabbit monoclonal YY1 antibody (ab-109237, Abcam) at 4°C overnight. Primary antibodies were diluted 1:200 using Dako antibody diluent solution (S0809, Dako). After two washes in Tris buffer (pH 7.4), sections were serially incubated with post Primary and Novolink Polymer (RE7150-K, Novolink Polymer Detection System, Leica) for 30 min. Immunoreactivity was visualized by adding diaminobenzidine (DAB) substrate for 3 min followed by counterstaining of nuclei with Mayer's hematoxylin. Whole slides were scanned, and the images were acquired at both DAB and hematoxylin channels using the TissueFAXS system (TissueGnostics GmbH). Hematoxylin staining was used as a master marker for cell identification on the basis of nuclear detection, and the average nuclear size, discrimination area, discrimination gray and background threshold for the master marker was specified using the HistoQuest software. The range of intensities of the master marker (hematoxylin) and the immunohistochemical stainings (i.e. DAB signals) were set by autodetection of the software. Regions of interest (ROIs) were defined as indicated, which were analyzed and quantified separately from the surrounding stromal areas. The general setups were done on a representative image. All images were analyzed with the same RGB (red, green, blue) color settings after adjustments (Hematoxylin; [94, 102, 145] and DAB: [126, 64, 64]). More than 45,000 ROIs were included in the analysis of each group of samples. The results are visualized in dot plot scattergrams and/or histograms. Cut-offs (to differentiate between positive and negative cells) and gates (to accentuate between cell populations) were set in the dot blots. Separation and counting in the cytoplasm and nucleus were performed according to the mean of the maximum and minimum values of hematoxylin master marker.

### Evaluation of the intrinsic disorder propensities of human YY1 and CP2c

Functional intrinsic disorder propensities of human YY1 (UniProt ID: P25490) and human CP2c (UniProt ID: Q12800) were analyzed by the computational platform D2P2 (http://d2p2.pro/) [53], which, in addition to showing disorder predisposition in a query protein by a set of established disorder predictors, such as PONDR^®^ VLXT, IUPred, PONDR^®^ VSL2B, PrDOS, ESpritz, and PV2, represents the location of functional domains, disorder-based binding sites and known sites of posttranslational modifications (PTMs).

### Analysis of the interactability of human YY1 and CP2c

Known information on the interactability of human YY1 and CP2c proteins was retrieved using the biological general repository for interaction datasets (BioGRID) that represents information on published protein interactions [54].

### Statistical analysis

Statistical analyses were performed using SPSS software 21.0 (SPSS). To determine optimal cut-off values for the expression of each protein, ROC curves were generated (sensitivity versus 1-specificity). AUC was calculated for each protein as well. Chi square test, Fisher's exact test, or linear by linear association test was applied to examine the associations between gene expression and clinicopathological characteristics. Kaplan-Meier analysis and log-rank test were used to calculate overall and disease-free survival curves. Multivariate Cox regression analyses were performed to evaluate independent prognostic factors for disease-free survival. For all tests, a *P* value less than 0.05 was considered statistically significant.

## SUPPLEMENTARY MATERIALS FIGURES AND TABLES


