# Supplementary material for: Diagnostic and prognostic relevance of CP2c and YY1 expression in hepatocellular carcinoma
Source: Oncotarget. 2017 Feb 17;8(15):24389–400. doi: 10.18632/oncotarget.15462 (PMC5421856; doi:10.18632/oncotarget.15462)
Supplement: Supplementary file 1 [file oncotarget-08-24389-s001.pdf]

Diagnostic and prognostic relevance of CP2c and YY1 expression in hepatocellular carcinoma

Supplementary Materials

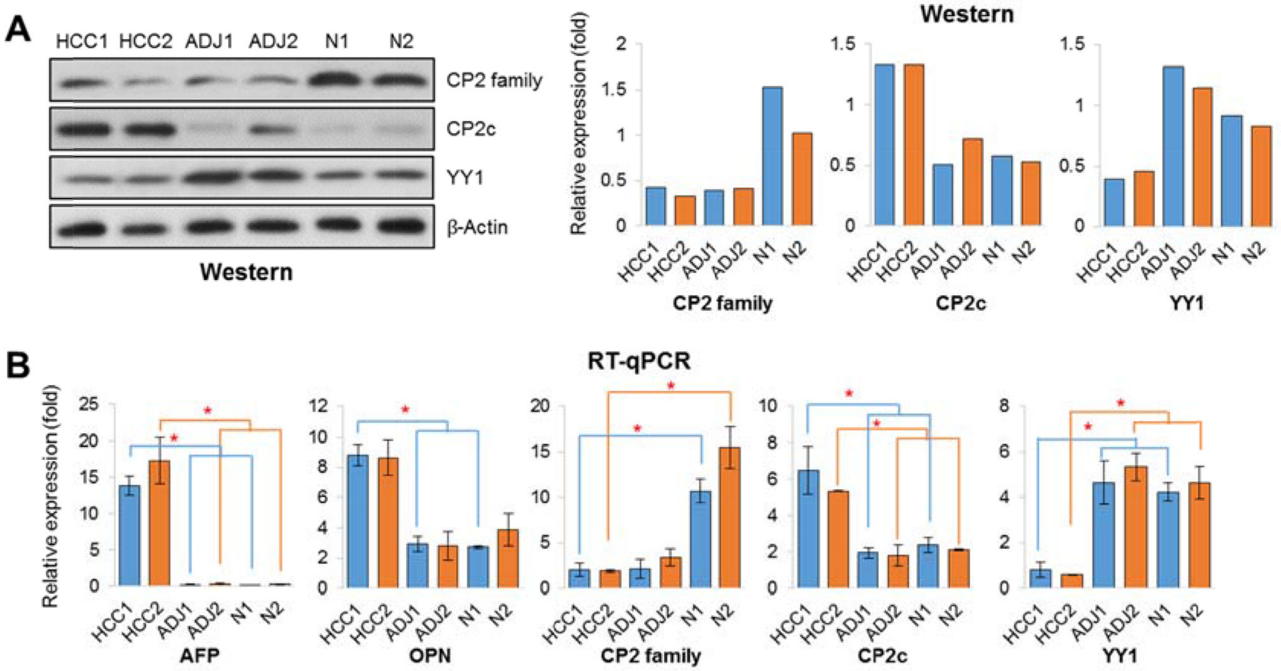

Supplementary Figure 1: CP2 family, CP2c, and YY1 expression in the ADJ and HCC tissues of the same patients at the mRNA and protein levels. Two HCC (HCC1 and HCC2) samples in the tissue assay shown in Figure 1 along with the matched ADJ noncancerous (ADJ1 and ADJ2) tissues derived from the same patents were subjected for analyses. Two normal liver (N1 and N2) samples were also used as controls. (**A**) Western blot showing expression of CP2 family, CP2c, and YY1 at the protein level (left). Relative expression of each protein to β-actin was shown in the right panel. (**B**) RT-qPCR analyses showing CP2 family, CP2c, and YY1 expression at the mRNA level. Expression of two HCC specific marker genes (α-fetoprotein (AFP) and osteopontin (OPN)) was also quantified. Values are shown as mean ± SEM; $n = 2$. $^*P < 0.05$.

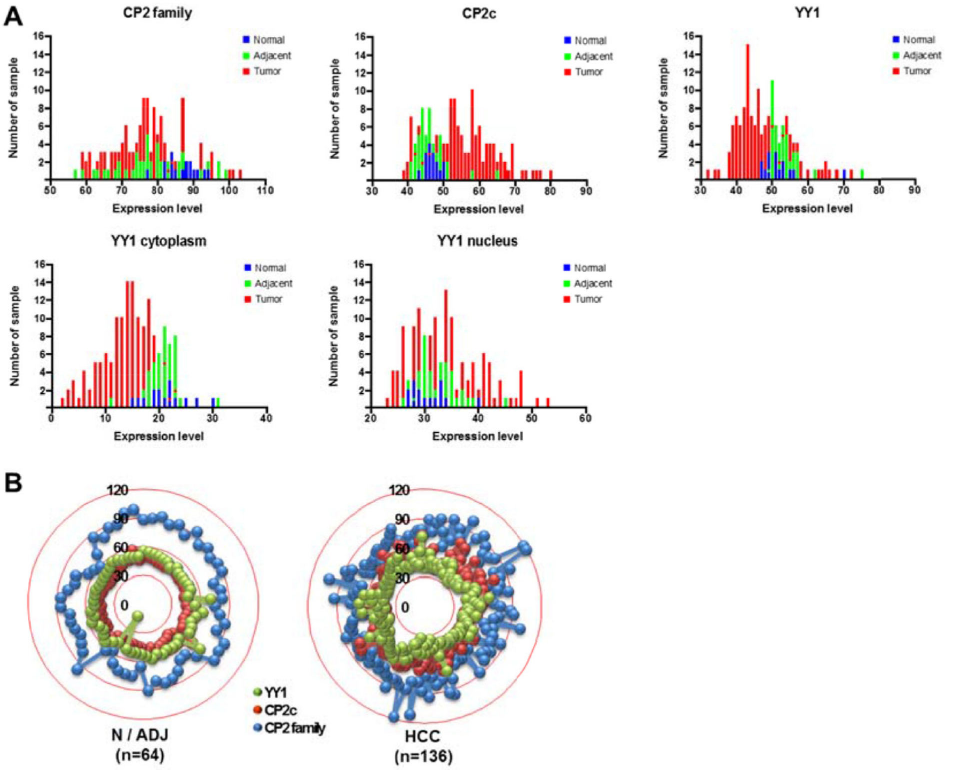

Supplementary Figure 2: Analysis of CP2 family, CP2c, and YY1 expression in liver tissues. (**A**) Immunoreactivity of CP2 family, CP2c, and YY1 (total YY1, cytoplasmic YY1, and nuclear YY1) proteins in TMA samples was measured using a TissueFAXS system. Frequency and expression level of each sample type (normal liver, adjacent noncancerous & HCC tissues) are shown. (**B**) Diagram showing the expression level and expression pattern of CP2 family, CP2c, and YY1 proteins in each type of samples in normal/adjacent noncancerous (N/ADJ) liver tissues and HCC tissues.

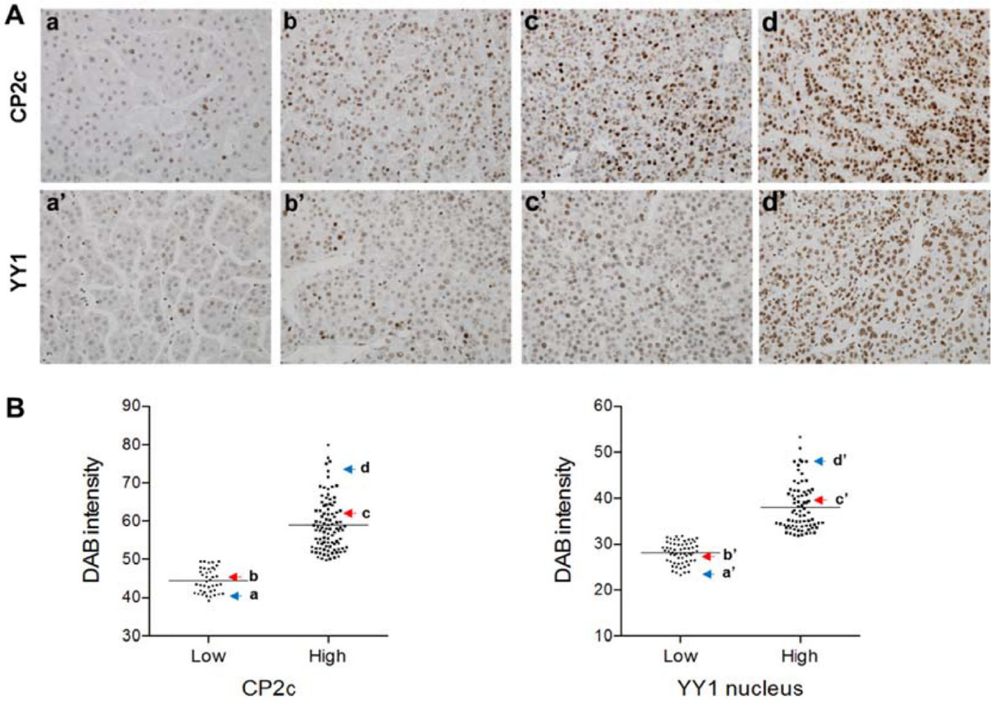

Supplementary Figure 3: Representative images showing the low or high expression of CP2c and nuclear YY1 proteins in HCC samples. Low expression and high expression groups of CP2c or nuclear YY1 were classified according to the cut-off value determined by ROC analysis of 116 HCC samples. (**A**) Two representative images of low expression group (a, b, a', b') and high expression group (c, d, c', d') in the panel B were shown. (**B**) Immunoreactivity (DAB intensity) of CP2c and nuclear YY1 of each HCC sample was dotted, and mean value of high or low expression group was shown in the graph.

SUPPLEMENTARY MATERIALS AND METHODS

RNA extraction from formaldehyde fixed, paraffin embedded (FFPE) tissue samples, cDNA synthesis, and RT-qPCR

Total RNAs were extracted from FFPE tissue sections as described previously [1]. Briefly, FFPE tissue samples were deparaffinized by addition of 1 mL xylene (TEDIA), followed by incubation for 3 min at 50°C, and centrifugation for 5 min at maximum speed. After the supernatant was discarded, the pellet was washed with xylene once again, and then washed twice with 1 mL absolute ethanol for rehydration. The proteins were degraded with 200 µL protease digestion buffer (20 mM Tris-HCl, pH 8.0, 1 mM $CaCl_2$, 0.5% SDS) containing 500 µg/mL protease K, followed by incubation for 3 h at 55°C. RNA was isolated using the TRIzol® reagent (QIAGEN) according to the manufacturer's protocol. The quality and quantity of the prepared RNA were examined by electrophoresis and spectrophotometry, respectively. cDNA synthesis and RT-qPCR were performed as described previously [2]. Primer sequences are as follows: hYY1 (5′-GAC ACC CTC TAC ATC GCC AC-3′ and 5′-TGT GGT CTC GAT GGT CTC CA-3′), hCP2c (5′-GGG TGC CAT TCC GAG TAC AA-3′ and 5′-GCC GAG TGT AAG TGC TCA GT-3′), hCP2 all (5′-TGG TGC AGG ACT TTG ATG C-3′ and 5′- GGG CAA TGC AAG GAC ATC AC-3′), hAFP (5′-AAA CTA TTG GCC TGT GGC GA-3′ and 5′-CAT GGC CTC CTG TTG GCA TA-3′), hOPN (5′-CAT ACA AGG CCA TCC CCG TT-3′ and 5′-TGG GTT TCA GCA CTC TGG TC-3′), and hβ-actin (5′-CTT CCA GCC TTC CTT CCT GG-3′ and 5′-CTG TGT TGG CGT ACA GGT CT-3′).

Protein extraction from FFPE tissue samples and western blot

Protein was extracted from FFPE tissue samples as described previously [3]. Briefly, FFPE samples were deparaffinized by incubation at room temperature in xylene for 10 min. After incubation, samples were pelleted by centrifugation for 3 min at maximum speed. After the supernatant was discarded, the pellet was washed with xylene once again, and then washed twice with 1 mL absolute ethanol for rehydration. The pellet was solubilized 200 µL of RIPA buffer, pH 7.6 (1 M sodium dihydrogen phosphate, 10 mM disodium hydrogen phosphate, 154 mM sodium chloride, 1% Triton X-100, 12 mM sodium deoxycholate, 0.2% sodium azide, 1 mM PMSF) containing 2% SDS, and the contents were incubated at 60°C for 2 h. After incubation, the tissue lysates were centrifuged at 12,000 g for 20 min at 4°C. The supernatants were collected and stored at −70°C until use. Western blot was performed as described previously [4], using rabbit polyclonal anti-CP2 family antibody (Cosmogentec), mouse polyclonal anti-CP2c antibody (610818, BD Biosciences), rabbit monoclonal YY1 antibody (ab-109237, Abcam), and goat polyclonal anti-β-actin antibody (sc1616, Santa Cruz). Proteins were visualized by chemiluminescence using an ECL system (Amersham-Pharmacia).

REFERENCES

1. Ma Z. Total RNA extraction from formalin-fixed, paraffin-embedded (FFPE) blocks. Bio-protocol. 2012; 2:e161.
2. Kim MY, Park J, Lee JJ, Ha DH, Kim J, Kim CG, Hwang J, Kim CG. Staufen1-mediated mRNA decay induces Requiem mRNA decay through binding of Staufen1 to the Requiem 3′UTR. Nucleic Acids Res. 2014; 42:6999–7011.
3. Ikeda K, Monden T, Kanoh T, Tsujie M, Izawa H, Haba A, Monden M. Extraction and analysis of diagnostically useful proteins from formalin-fixed, paraffin-embedded tissue sections. J Histochem Cytochem. 1998; 46:397–403.
4. Guo H, Liu W, Ju Z, Tamboli P, Jonasch E, Mills GB, Tsavachidou D. An efficient procedure for protein extraction from formalin-fixed, paraffin-embedded tissues for reverse phase protein arrays. Proteome sci. 2012; 10:56.

Supplementary Table 1: Receiver operating characteristic curve data

Tested proteins	AUC	95% CI	Cut off	sensitivity	specificity	*P* value
	**Panel A (N vs HCC)**					
CP2 family	0.205	0.128–0.282	94.147	0.081	1.000	< 0.01
CP2c	0.791	0.724–0.859	49.819	0.704	1.000	< 0.01
YY1	0.334	0.216–0.452	55.687	0.133	0.938	0.030
YY1 cytoplasm	0.161	0.035–0.287	0.647	1.000	0.063	< 0.01
YY1 nucleus	0.657	0.543–0.772	33.843	0.444	0.938	0.040
	**Panel B (Non-survivor vs Survivor in DFS)**					
CP2 family	0.619	0.526–0.713	76.020	0.672	0.535	0.017
CP2c	0.696	0.606–0.785	50.519	0.875	0.493	< 0.01
YY1	0.451	0.352–0.550	50.555	0.344	0.732	0.330
YY1 cytoplasm	0.408	0.307–0.510	19.104	0.188	0.930	0.067
YY1 nucleus	0.553	0.455–0.650	31.946	0.656	0.535	0.292

AUC, Area under the curve; CI, Confidence interval; N, Normal liver tissue; HCC, Hepatocellular carcinoma tissue.

Supplementary Table 2: Correlation of CP2c expression and nuclear YY1 expression in different groupings of historical grade and AJCC stage in HCC

Characteristics		CP2c		*P* value (x^2 test)	YY1 nucleus		*P* value (x^2 test)
		Low	High		Low	High	
Histological grade				**0.024**			0.234
	G1 & G2	23 (39.7%)	35 (60.3%)		29 (50.0%)	29 (50.0%)	
	G3 & G4	17 (21.8%)	61 (78.2%)		31 (39.7%)	47 (60.3%)	
				**0.013⁺**			0.098⁺
	G1	6 (60.0%)	4 (40.0%)		7 (70.0%)	3 (30.0%)	
	G2	17 (35.4%)	31 (64.6%)		22 (45.8%)	26 (54.2%)	
	G3	15 (21.7%)	54 (78.3%)		28 (40.6%)	41 (59.4%)	
	G4	2 (22.2%)	7 (77.8%)		3 (33.3%)	6 (66.7%)	
AJCC stage				**0.005**			**0.002**
	I	28 (40.0%)	42 (60.0%)		40 (57.1%)	30 (42.9%)	
	II~IV	12 (18.2%)	54 (81.8%)		20 (30.3%)	46 (69.7%)	
				**0.007⁺**			**0.019⁺**
	I	27 (39.7%)	41 (43.2%)		39 (57.4%)	29 (42.6%)	
	II	9 (20.0%)	36 (80.0%)		13 (28.9%)	32 (71.1%)	
	III	2 (40.0%)	3 (60.0%)		2 (40.0%)	3 (60.0%)	
	IV	1 (6.3%)	15 (93.8%)		5 (31.3%)	11 (68.8%)	

P value < 0.05 marked in bold font shows statistical significance.

⁺Linear by linear association test.

Supplementary Table 3: Clinical and pathological characteristics of HCC patients

		Included	Excluded	Total
		Number	Number	Number
Age		136	0	136
	< 57	70		
	≥ 57	66		
Gender		136	0	136
	Female	33		
	Male	103		
HBsAg status		134	2	136
	Negative	33		
	Positive	101		
Histological grade		136	0	136
	G1	10		
	G2	48		
	G3	69		
	G4	9		
AJCC stage		134	2	136
	I	68		
	II	45		
	III	5		
	IV	16		
Tumor size		134	2	136
	< 5 cm	97		
	≥ 5 cm	37		
Small vessel invasion		135	1	136
	Absent	78		
	Present	57		
Large vessel invasion		135	1	136
	Absent	119		
	Present	16		
Vessel invasion		135	1	136
	Absent	77		
	Present	58		
Perineural invasion		135	1	136
	Absent	131		
	Present	4		
Focality		135	1	136
	Single	112		
	Multiple	23		
Disease free survival (DFS)		116	20	136
	Survivor	53		
	Non-survivor	63		
Overall survival (OS)		115	21	136
	Survivor	59		
	Non-survivor	56		
